# Stimulus Onset Hub: an Open-Source, Low Latency, and Opto-Isolated Trigger Box for Neuroscientific Research Replicability and Beyond

**DOI:** 10.3389/fninf.2020.00002

**Published:** 2020-02-06

**Authors:** Charles E. Davis, Jacob G. Martin, Simon J. Thorpe

**Affiliations:** Centre de Recherche Cerveau et Cognition (CerCo), CNRS-Universitè Toulouse 3, Toulouse, France

**Keywords:** opto-isolation, stimulus tracking, psychophysics, open-source, arduino, EEG, SEEG, ECoG

## Abstract

Accurate stimulus onset timing is critical to almost all behavioral research. Auditory, visual, or manual response time stimulus onsets are typically sent through wires to various machines that record data such as: eye gaze positions, electroencephalography, stereo electroencephalography, and electrocorticography. These stimulus onsets are collated and analyzed according to experimental condition. If there is variability in the temporal accuracy of the delivery of these onsets to external systems, the quality of the resulting data and scientific analyses will degrade. Here, we describe an approximately 200 dollar Arduino based system and associated open-source codebase that achieved a maximum of 4 microseconds of delay from the inputs to the outputs while electrically opto-isolating the connected external systems. Using an oscilloscope, the device is configurable for the different environmental conditions particular to each laboratory (e.g., light sensor type, screen type, speaker type, stimulus type, temperature, etc). This low-cost open-source project delivered electrically isolated digital stimulus onset Transistor-Transistor Logic triggers with an input/output delay of 4 μs, and was successfully tested with seven different external systems that record eye and neurological data.

## Introduction

Multimodal experimentation in neuroscience research is becoming common practice. Not only can inputs be multimodal (e.g., auditory stimuli, visual stimuli, somatosensory stimuli, etc.), but the output devices to which these input triggers are sent are also potentially multimodal (e.g., EEG+eyetracking, EEG+fMRI, EcoG+SEEG+eyetracking, etc.). Multimodal recordings from multimodal inputs pose technical challenges that relate to electrical interference, crosstalk, and temporal precision of stimulus onsets. The experiments in our neuroscience lab generally explored humans' capabilities to do continuous ultra-fast face and sound detection (Martin et al., 2017, 2018a,b). The experiments operated at high speeds, and we needed to align the visual and auditory stimulus onsets with various combinations of recording equipment such as eye tracking, electroencephalography (EEG), stereo electroencephalography (SEEG), and electrocorticography (ECoG) (Fried et al., 2014). The delay for detecting and delegating the recorded triggers to external equipment is an important factor due to the temporal nature of the recorded neurological signals (Plant et al., 2004). Inaccurate stimulus timing can cause both Type I (false positives) and Type II errors (false negatives). As a solution to the problem of detecting analog and digital events and delivering them to different systems without significant delay, we describe an inexpensive and ultra-low latency device that we designed and built to detect and deliver visual and auditory stimulus onsets to multiple external data acquisition systems. The code and design of the system are further described at the project's website (https://stimulusonsethub.github.io/StimulusOnsetHub/).

**Figure 1:**
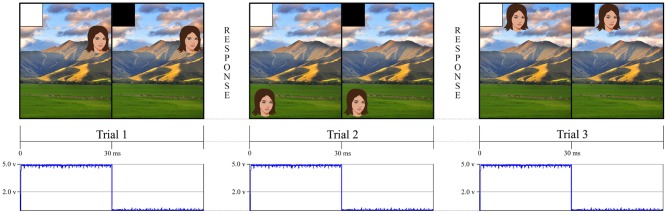
This figure is an example of a simple experiment using an eye tracker and a stimulus computer. The eye tracker followed the eyes as participants made continuous saccades toward different face targets. The stimulus screen presented a white square in the upper left hand corner for 30 ms to indicate when the trial had begun and then a black square, in the same location, until the stimulus system was ready to present another trial. Our stimulus tracking device used a photodiode that was affixed to the screen to detect when the white square appeared. This allowed us to independently verify when an event happened rather than relying on the stimulus presentation computer to tell us when the event happened through potentially inaccurate GPU timestamps. For the landscape photo attribution please see Learning and learning (2009). Available online at: https://commons.wikimedia.org/wiki/File:NZ_Landscape_from_the_van.jpg.

**Figure 2:**
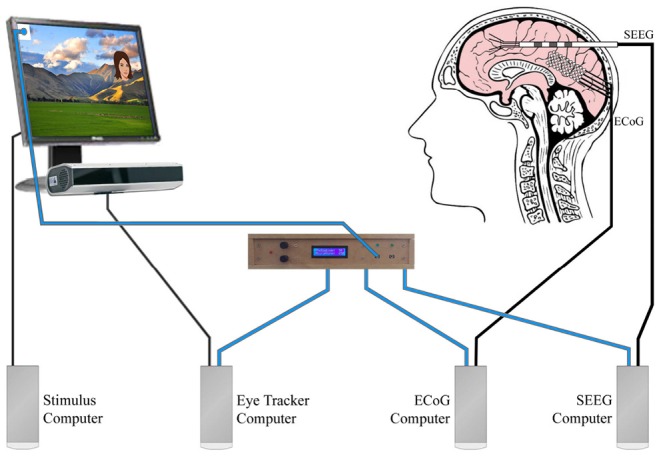
This figure is based on the same experiment described in Figure 1. This multidevice experiment example used simultaneous eye tracking, ECoG, and SEEG. Blue wires carried the analog photodiode signals to our stimulus onset hub (center). The stimulus onset hub continuously checked the analog signals to see if they crossed a user-specified threshold. If they did, the stimulus onset hub transformed the analog signal into a digital opto-isolated TTL signal that was then sent to the different external recording systems (eye tracker computer, ECoG Computer, SEEG computer). Decoupling the systems with opto-isolators prevented potential interference that could arise due to differential grounding and electrode proximity. For the landscape photo attribution see Learning and learning (2009). Available online at: https://commons.wikimedia.org/wiki/File:NZ_Landscape_from_the_van.jpg.

Our goal was to design and build a robust and affordable device that would perform its duties with minimal delay while minimizing electrical crosstalk between the different externally connected systems. We were concerned about potential data quality loss associated with noise coming into our EEG, SEEG, ECoG, and eye tracking systems due to potential ground loops and crosstalk created at the different connections to the trigger hub. When multiple recording systems were connected at the source of the stimulus detector, ground loops between these systems created unwanted noise that contaminated the recorded signals of interest. Thus, we designed the device to opto-isolate the different recording systems from each other in order to reduce noise and thereby improve data quality. Next, we interfaced the device with the photodiode and microphone sensor devices to accurately timestamp stimulus onsets within the experiments (see Figure 1). The light and sound detectors bypassed additional latencies introduced by the stimulus computer, monitor, operating system, experimental application, graphics card, sound card, speakers, etc. (see Figure 2) (Plant et al., 2002).

We requested quotes for stimulus trackers from three different companies and received prices ranging from 1,645 to 4,488 US dollars. Each company offered add-ons to interface with more than one external recording device, but these were also rather expensive. For example, one company offered an adapter for our BioSemi Active Two (BioSemi, Amsterdam, Netherlands) data acquisition system for 125 US dollars along with another adapter for our Eyelink 1,000+ eye tracker (SR Research, Ottawa, ON, Canada) for 55 US dollars. Another company charged 395 US dollars for all three connections that we needed. No company seemed to offer opto-isolation, but we couldn't be sure without opening each device. Instead of purchasing a commercial system with unknown schematics, we spent about 200 US dollars and made the opto-isolated device ourselves.

We used the Arduino platform, which has previously been used in a variety of laboratory based experiments for behavioral research (D'Ausilio, 2012) and neural research (Osman et al., 2012). We tested and optimized the input and output delays according to our particular experimental conditions and hardware. The project ultimately saved us thousands of dollars over other commercial systems that were not opto-isolated, while also improving performance. The device, in conjunction with an oscilloscope, allowed us to find additional latencies introduced by other experimental devices and improved the quality of our data by eliminating electrical crosstalk and ground loops between the external connected devices. After optimization, the input/output lag of the device had a maximum delay of only 4 μs.

## Materials

Here we describe our design and why we made different design decisions. We describe in detail how we realized and built this device with circuit diagrams, Arduino pin maps, benchmarking code, and Arduino C code. The design and code are the end result of 2 years of research with many different prototypes. We built the final hardware's enclosure in about 4 hours and wired the circuit in about 30 min.

### Device Design

The Arduino Mega 2560 Rev3 is an open-source microcontroller board based on the ATmega2560 microcontroller. We chose the Arduino Mega because it offered the most ports in the Arduino family. It has 54 digital input/output pins with 14 of these pins available for use as pulse width modulation (PWM) outputs, 16 analog inputs, a 16 MHz crystal oscillator, a USB connection, and a power jack. The Arduino can be powered by a computer or a USB cable, but, according to the documentation, if supplied with <7 V, the 5 V pin may supply <5 V, and the board may become unstable. We found this to be true when we tested our device. To overcome this problem, we chose a 12 V, 1 amp AC-to-DC adapter (wall-wart) with a 2.1 mm center-positive barrel connector.

We chose breakout boards with screw terminal blocks to provide a more robust development platform for easy prototyping and hassle-free testing with an oscilloscope. We avoided soldering, as well as desoldering, as much as possible. We also avoided buying expensive crimping tools to make connections such as Dupont and Japan Solderless Terminal (JST) wire connections. Additionally, we used WAGO brand splicers for further ease of use. We used multicolored, 22 American Wire Gauge (AWG) wire from Adafruit Industries that was the exact gauge of the Arduino ports. We found that the wires with Dupont connectors that came with most Arduino starter kits did not feel secure in the Arduino Mega ports, but the Adafruit wires definitely seated better.

In order to confront the problems of noise due to electrical crosstalk and ground loops, we used a dual channel opto-isolator break out board made by SparkFun Electronics to isolate the input side of our stimulus tracker from the output side. It is important to note that, since the inputs were opto-isolated from the outputs, the output side needed a power supply. Fortunately, all but one of the recording devices we tested for compatibility provided several power and ground pins. This recording device did not provide a power source because it had only BNC input connections. To work around this problem, we used a battery pack with an on/off switch that produced approximately 4.5 V to power the output side of the opto-isolator for the BNCs. It was necessary to solder screw terminal blocks for these break out boards.

We used a 1602 liquid-crystal display (LCD) screen with an I2C bus from SunFounder for the graphical user interface. In order to display the input device sensitivity levels that were used to control the analog threshold levels needed to send a digital TTL output, we used an LCD with a pre-attached I2C bus which helped reduce wired connections to the Arduino. We used two rotary encoder break-out boards with plastic knobs to control input device threshold levels. For trigger and power indicator lights, we used red, blue, and green LEDs. We soldered the LEDs to their respective 100 ohm resistors and wires. We used audio jack break out boards with pre-attached screw terminal blocks from Gravitech Electronic Experimental Solutions and screw terminal block to male 3.5 mm audio connectors for wiring the photodiode and connecting to the audio jacks. We used version two of an analog ambient light sensor from DFRobot that had a published light ramp up time of 15 μs DFRobot.

For the output side of the device, we purchased D-subminiature (DB) 25 and 37 breakout boards from CZH-LABS with pre-attached screw terminal blocks. We used electromagnetically shielded DB25 and DB37 cables with adapters to connect the outputs to the different recording systems. We chose Bayonet Neill-Concelman (BNC) connector breakout boards, also from Gravitech Electronic Experimental Solutions, with pre-attached screw terminal blocks.

A future version of this project could eliminate the Arduino and have a custom printed circuit board. However, the downside to building a fixed PCB is that it would lose some of the plug-and-play flexibility that the Arduino platform provides. In addition, a better enclosure material could be chosen. We used Medium Density Fiber board to make our enclosure for ease of prototyping (i.e., Figures 5–7). One could use a 3D printer to print the enclosure in plastic. Perhaps the best choice would be to use an electrically isolating material like hard rubber (Das et al., 2001).

### Code Design

We programmed the hardware in the Arduino C Language and made it freely available on the Internet and named it StimulusOnsetHub (Martin and Davis, 2019). On the GitHUB page (https://stimulusonsethub.github.io/StimulusOnsetHub/), there is also a hardware list and more detailed information for how to build the device along with suggested tools, computer hardware, and other components for building highly accurate behavioral experiments. In the code, the “setup()” function is called once when the Arduino powers up, and sets up all pin modes and interrupts for the different connections. The “loop()” function is called repeatedly while the box is running and handles:

- Reading the photodiode and microphone input levels,
- Sending digital TTL output signals if the analog/digital input levels crossed the user-specified threshold level for each input, and
- Optionally updating the LCD if an interrupt from one of the rotary encoders was triggered.

The performance of the “loop()” function largely determined the temporal delay between the inputs and outputs of the box, aside from any external delays caused by other factors. We used interrupts for the rotary encoders to avoid the performance reduction that would occur if the rotary knobs' positions were read each time during the loop. Aside from this early optimization, we tried to avoid other premature optimizations: “…the root of all evil (or at least most of it) in programming (Knuth, 1974).” That is, at the end of the software and hardware design, we built compiler switches in the code to test the effects of different combinations of optimization choices with the oscilloscope. We explored several different on/off binary compiler options, such as:

- LIGHTSENABLED: Optionally turn off the LCD and LED lights of the box.
- DIGITALWRITEFAST: Optionally use the digitalWriteFast library (Watterott, 2017).
- DIGITALMICROPHONE Optionally use a digital microphone.
- ADDA: Optionally support an analog daughterboard.
- FASTADC: Optionally set different pre-scale methods on potentially connected analog to digital converters.
- DEBUG: Optionally enable debug statements to be sent to the console. This compiler option is for debugging and verifying the software and hardware.

**Figure 3:**
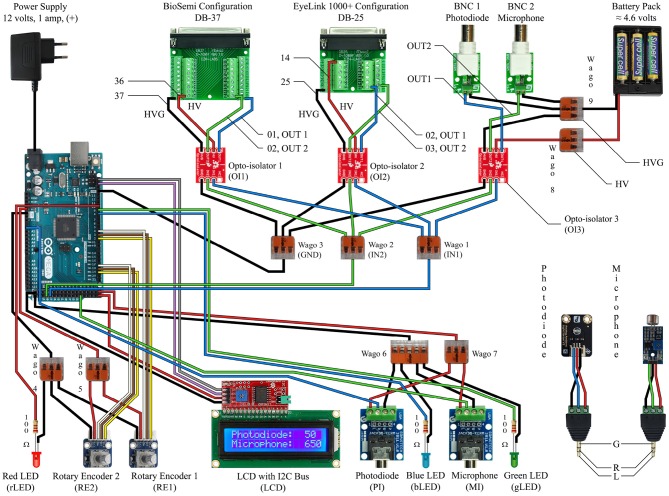
This circuit diagram describes the device we made to work with our EEG system and eye tracker, and it should be read in conjunction with the Arduino Mega 2560 Rev3 Pin Map (reference Figure 4). The red and black wires correspond to power and ground. The white, brown, and yellow wires are only for the rotary encoders. The gray and violet wires correspond to the SCL and SDA connections for the LCD screen. The blue wires indicate the path of the photodiode input signal to the output signal as well as the blue indicator light. The green wires indicate the path of the microphone input signal to the output signal as well as the green indicator light.

**Figure 4:**
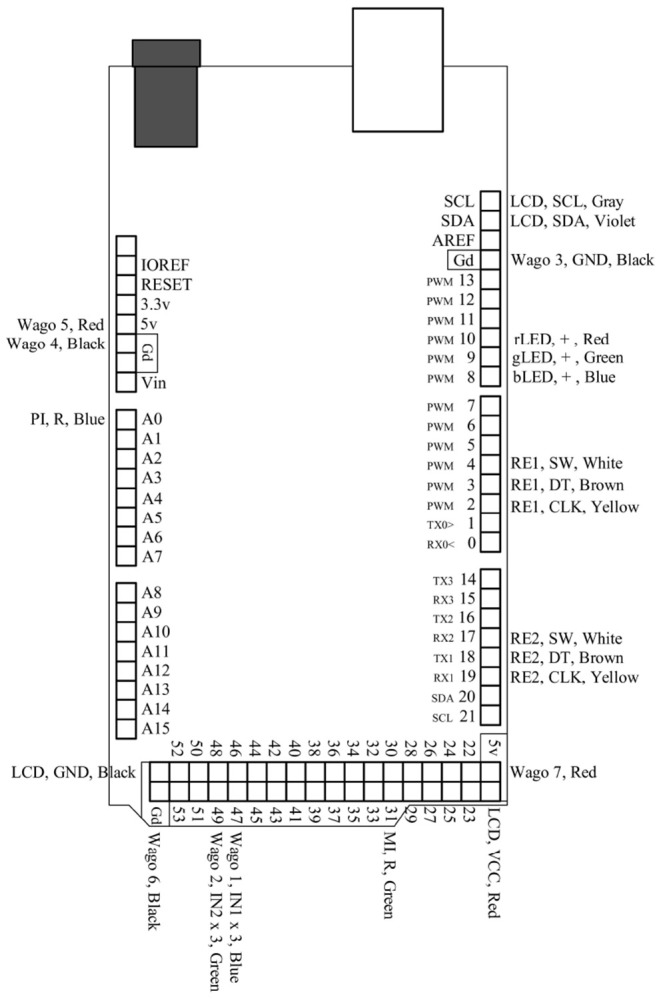
This is a pin map for the Arduino Mega 2560 Rev3. All used ports are labeled and correspond to their respective connections in Figure 3.

We encountered a limitation with the Arduino in that we could only use one of the analog inputs at a time because of pre-existing time constraints on switching between the reading of different analog channels. The Arduino could only check the analog input rail one analog input at a time. For example, if a photodiode was hooked up to analog input A0 and an analog microphone was hooked up to analog input A1, the Arduino could not read the signal from A0 and the signal from A1 at the exact same time. Instead, there was a hardware determined delay between the reading of two different analog channels. Thus, we included a second option in the code (ADDA) to allow the use of a separate analog to digital daughter board. This setup allowed us to control the threshold of an analog microphone, and was faster than reading from A0 and then switching to A1, but still included a hit in performance as the code in “loop()” had to do more work. Ultimately, we decided to use a digital microphone with an integrated potentiometer that was tuned to the most sensitive setting that still reliably detected sound. All wiring diagrams shown in this paper correspond to using an analog photodiode and a digital microphone (i.e., Figures 3, 4).

**Figure 5:**
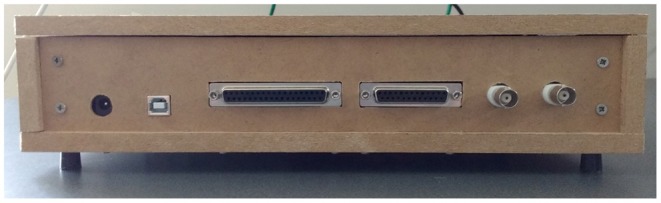
The back panel of the device. From left to right, the connections are: 2.1 mm barrel connector for the power supply, USB port for updating the software, DB37, DB25, photodiode output BNC connector, and microphone output BNC connector.

## Results

We measured the processing delay through the box using the digital inputs. To perform this test, we used the oscilloscope to average 30 iterations of a pure-tone sound wave that was played through the speaker. The digital output of the microphone was then used as the input to both the stimulus onset hub and oscilloscope. As verified with the oscilloscope, the digital latency through the box had a maximum input/output delay of only 4 μs (**Figure 8A**).

To test the effects of different thresholds on the temporal precision of the TTL output signals, we wrote a benchmarking tool in Matlab using Psychtoolbox (Brainard, 1997). The Psychtoolbox program alternately painted 500ms black and white squares on an ASUS PG278Q computer screen with a refresh rate of 120 Hz to trigger the photodiode. This LCD computer screen has been shown to give accurate results, if configured with ULMB mode enabled (Ultra Low Motion Blur) (Zhang et al., 2018). We did four threshold tests, two with ULMB enabled (with thresholds 10 or 180), and two with ULMB disabled (with thresholds of 10 or 180). After launching the benchmarking tool, it cycled through 30 on/off blips. We then used the oscilloscope to average the latency between the rising edges of the input signals and the rising edges of the output signals of the stimulus onset hub. The results indicated that the threshold setting can drastically affect the temporal accuracy in both modes (**Figures 8B,C**).

In more detail, we used the front 3.5 mm audio port to track the photodiode input signal and the DB25 connector on the back of the device to track the output signal. We placed a short piece of wire in the screw terminal block port for the photodiode input signal and another short piece of wire into the ground connection on the Arduino. Next, we inserted short pieces of wire into the screw terminal block ports of the DB25 for the output signal and the ground. On the oscilloscope, we attached 1 hook-tip probe onto the channel 1 connection and another onto the channel 2 connection. In order to set up this experiment, we first placed the hook-tip probe for channel 1 on the testing point on the photodiode screw terminal block and the alligator clip ground to the ground connection on the Arduino. To acquire the output signal, we placed the hook-tip probe for channel 2 on the signal testing point on the DB25 screw terminal block and the alligator clip ground to the ground connection on the DB25 screw terminal block. The Eyelink eye tracking system provided power and ground for the output side of the opto-isolator over the DB25 connection.

We computed all timing delays through the box using the Tektronix TBS1064 digital oscilloscope. Before each experiment, we allowed the oscilloscope to warm up for 20 min as suggested by the user manual. We also made sure to perform low-frequency compensation on all probes prior to testing. Additionally, we used the Do Self-Calibration utility, which was provided in the oscilloscope Utility menu, to account for any temperature variation that may have occurred in our testing environment since the last calibration. In order to compare the two signals, we moved the vertical position of channel 2 (the TTL output after the opto-isolator) on top of channel 1 (the analog photodiode input), and set the oscilloscope to averaging mode. We used channel 2 as the triggering line, set the trigger level to 3 volts, and set the oscilloscope to detect the rising edge of the signal.

The input and output delay of the box was at most 4 μs. The photodiode that we used had a reported onset delay of 15 μs. Thus, the total latency to detect and transmit an opto-isolated digital TTL signal to multiple external recording devices was around 19 microseconds for the particular photodiode that we used. We did not try to find better photo-detectors or optimize the code any further because we had already achieved a temporal accuracy that was adequate for our purposes. Sampling rates for neural and eye tracking systems are on the order of $\frac{1}{1,000}$ to $\frac{1}{10,000}$ seconds, which is respectively 52.6 to 5.3 times slower than a $\frac{19}{1,000,000}$ second delay. So, even if we had achieved a better precision, it wouldn't have helped with the neural and eye tracking technologies that were currently available.

We started our compatibility tests with the Eyelink eye tracker system and then with the BioSemi EEG recording system, but our device will work with any device that can receive a TTL input. Additionally, we tested other devices for compatibility, including the Red and IViewX line of eye trackers from SMI (SensoMotoric Instruments GmbH, Teltow, Germany), and the Blackrock (Blackrock Microsystems, Inc., Salt Lake City, UT, USA), Neuralynx (Neuralynx, Inc., Boseman, MT, USA), and Micromed (Micromed SAS, Lyon, France) lines of brain recording devices. One drawback of the do-it-yourself approach was that a given design was not completely plug and play across the 7 different recording systems because each had idiosyncratic connection types. Thus, depending on the desired device with which to interface, we found that it was sometimes necessary to spend time going through user manuals, pin out schematics, and internet forums to find out how to make each individual device work. Other times, we needed a voltmeter to map out every pin connection on a device. However, these are unavoidable realities of interfacing TTL signals with different types of modern hardware.

The design should be used in conjunction with an oscilloscope during calibration because experimental conditions such as the particular sensors used and even the environmental temperature can affect sensor readings and sensitivities (Prokeš, 2007). The more precisely we set the threshold levels for the inputs to the Arduino device, the lower the latency between the input and output signals through our device (**Figures 8B,C**). This occurred because the photodiode input was an analog signal whereas the external recording devices required digital inputs. For example, **Figure 8B** shows how sensitive thresholds like 10 were associated with a more precisely aligned output trigger (with respect to the very beginning of the analog photodiode onset) compared to less sensitive thresholds like 180 which were associated with delays of about 8 ms. When the monitor was in the ULMB mode (Ultra Low Motion Blur), the delays introduced by a less sensitive threshold of 180 were about 100 μs (**Figure 8C**). When we enabled ULMB, set the monitor's brightness to 100 and contrast to 75, and set the threshold on the box to 10, we observed a 10 μs delay between the start of the analog photodiode onset and the output trigger. Thus, the trigger threshold level was a critical component that was dependant on environmental conditions and had to be verified with an oscilloscope.

## Discussion

Combinations of different types of stimuli (e.g., auditory, visual, manual reactions) and external neural recording devices (e.g., EEG, ECog, Eyetracking) complicate the coordination of sending stimulus onsets to different systems both electrically and temporally. When multiple systems are connected at the source of the stimulus processor, electrical interference can occur between the external systems, leading to undesirable crosstalk which can degrade the quality of the data. Commercial products exist to deliver stimulus triggers to external devices, but we were unable to find any that electrically isolated the connected systems or that performed with the ultra-fast processing that we achieved in the device that we built. The DIY aspect of this project gave us more fine tuned control than using expensive commercial trigger devices. Such a modular, open source approach provides a solid foundation for anyone else to build upon for their own needs. To our knowledge, we have included everything needed for accurately marking a stimulus onset in an experiment and delivering it to multiple external recording systems in an opto-isolated manner. We tested seven different external recording devices for compatibility with our trigger box: three different eye trackers (from two different companies), three different neural recording devices (from three different companies), and one EEG system. We also saved thousands of dollars that was instead put toward other aspects of the overall research project. Although we designed this device with neuroscientific experiments in mind, it can be easily adapted to other scientific fields where ultrafast temporal accuracy and signal quality are important.

How big of a problem does this device solve? There is currently a replication crisis in many fields of neuroscience and psychology, with some estimates claiming up to 64% of research in psychological science is not reproducible (Kerr, 1998; Wicherts et al., 2006; Wager et al., 2009; Simmons et al., 2011; Button et al., 2013; Gelman and Loken, 2013; Lindquist and Mejia, 2015; Crandall and Sherman, 2016; Etz and Vandekerckhove, 2016; Anderson et al., 2017; Johnson et al., 2017; Luck and Gaspelin, 2017; Munafò et al., 2017; Poldrack et al., 2017; Szucs and Ioannidis, 2017; Georgescu and Wren, 2018; Ioannidis, 2018; Zhang et al., 2018). Three common problems that have been suspected to cause the failure to replicate such studies are small sample sizes, “hypothesizing after the results are known,” and “p-hacking.” However, if 64% of all psychological science is not reproducible, what percent of this 64% is due to errant stimulus timing or unshielded systems? We are unaware of any meta-studies that include such an estimate. However, the inspiration for this work came from our own personal experiences with the replication crisis, due to *both* Type I and Type II errors, from several members of our own laboratory and others. For example, just a few years ago, a bug in a widely-used EEG manufacturer's system delivered wildly inaccurate temporal triggers. The problem existed for a long time and was so significant that it ultimately led to many inter-lab replication troubles and the retraction of several papers. One can show with a simple thought experiment how both Type I (false positive) and Type II (false negative) errors can arise from inaccurate timing. Consider, for example, a situation where the timing of triggers is late by 50 ms in an experiment on visual EEG signals. Thus, an electroencephalographic potential that was erroneously thought to occur at 150 ms, would lead to a false positive error at 150 ms because it would create a significant potential where there actually was none. In addition, and at the same time, it would also create a false negative error at 100 ms because the significant potential at 100 ms would not be found, when it in fact existed. Thus, shifting signals in time can create both false positive and false negatives. In addition, opto-isolation helped avoid false positive and negative errors because it improved the signal to noise ratio. Avoiding both of these types of errors is very important from the perspective of someone who is trying to study the brain in a reproducible manner.

There are several improvements that can be made to our design. One functional improvement would be to provide an internal oscilloscope or an automatic input/output delay calculation function to tune the input level thresholds to minimize the input/output delay. One could splice the signal output from the photodiode input two ways from within the box. One direction would go to the Arduino as normal, and the other would go to an additional BNC breakout board on the front of the device for hooking up one channel of an oscilloscope. A poor splice can affect both the voltage and current levels. Thus, the best photodiode threshold level might change when splicing the photodiode toward both the oscilloscope and the Arduino. To solve this problem, one could leave the oscilloscope connected for the experiments to be sure of the timing delay and the best settings for the input thresholds. Therefore, one could essentially have an online oscilloscope during each experiment to store verification data for the trigger timing for each trial. Another functional improvement could be to use fiber optic transmitters and receivers for the output signals to the data recording devices. This would eliminate the need for the opto-isolators, which did introduce a very small delay into the system. Using fiber optics would also allow further noise reduction and make cable management easier compared to expensive and cumbersome electromagnetically shielded DB25 and DB37 cables. Physical switches for turning off the indicator lights during the experiment could be added to increase the speed of the code and control the room's lighting during experiments. We considered using the push button function of the rotary encoders to accomplish this, and the code supports this function. However, it is also necessary to consider that checking physical switches in the code can take up processing time and increase delays.

This project is open source, inexpensive, configurable, flexible, reduces experimental noise, and improves temporal reproducibility. Based on this paper, the project can be reproduced easily and inexpensively. As we have open-sourced the design and software (Martin and Davis, 2019), anyone can improve or use the design however they choose. One could simply use a wiring scheme as described in this paper, or they could have a printed circuit board made once their augmented design is finished. Anyone can make their own or have a professional company make one for them. Maybe, owing to its low cost, open codebase, and opto-isolated and ultra-low latency processing times, the device could provide a verifiable standard for any experimental research project that will be published and requires verification of ultrafast and noiseless delivery of stimulus onsets to multiple recording systems.

## Data Availability Statement

The datasets generated for this study are available on request to the corresponding author.

## Author Contributions

**Figure 6:**
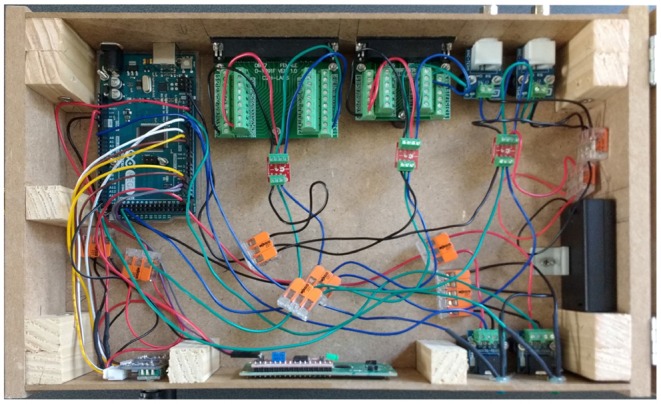
Overhead view of the device showcasing the internal wiring. This is a different configuration than that of Figure 3, and is for presentation purposes only. The connections for the rotary encoders and the LCD are different here. To build the device, follow the circuit diagram, Arduino pin map, and code.

**Figure 7:**
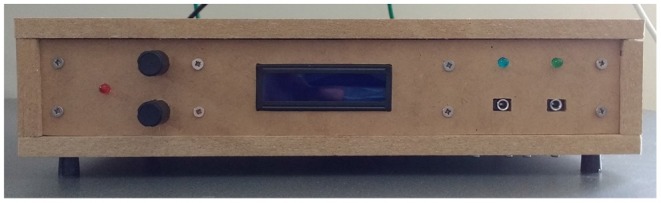
Front panel and user interface for the device. From left to right, the elements are: red power indicator light, rotary encoder adjustment knobs, LCD, photodiode, and microphone input jacks with corresponding blue and green indicator lights.

**Figure 8:**
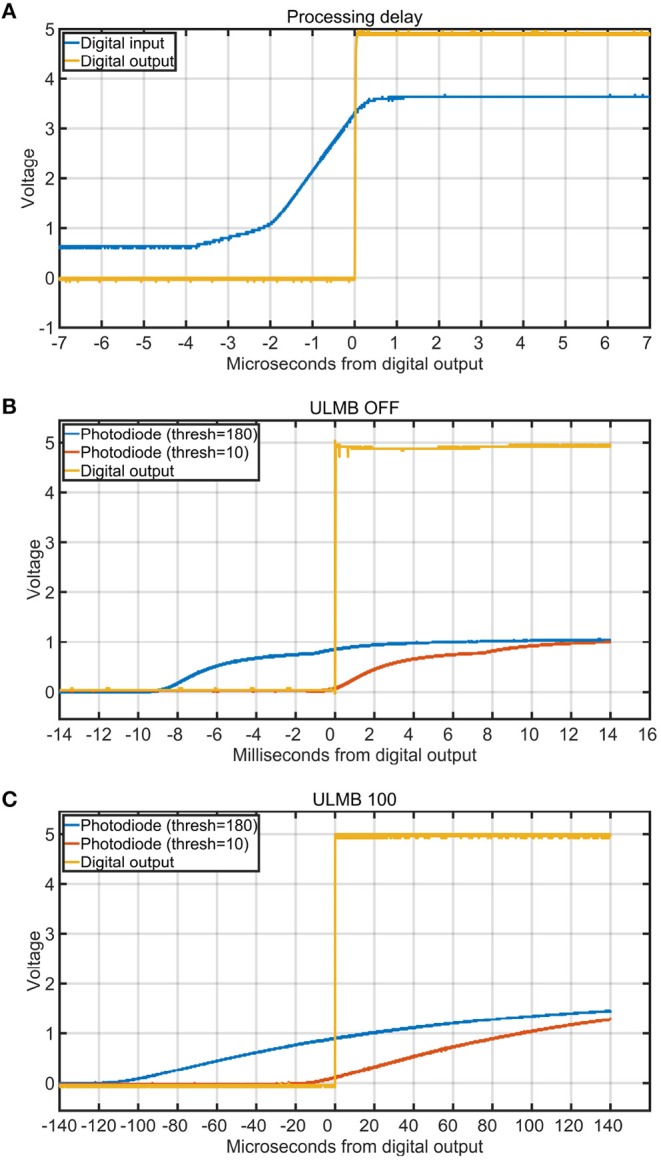
Processing delays and the influence of trigger threshold on stimulus onset timing. Each curve is the average of 30 iterations. **(A)** Delay in **micro**seconds between a digital input (blue) and the digital output of the box (yellow) for one channel. **(B)** Delay in **milli**seconds between the digital output (yellow) and the start of the analog photodiode signal onsets for different triggering thresholds (blue = 180 and red = 10). The monitor was set to 100 brightness, 75 contrast, and ULMB mode (Ultra Low Motion Blur) was turned off. This figure shows how a threshold of 180 introduced a delay of more than 8 ms, whereas a threshold of 10 had very good temporal precision. **(C)** Delay in **micro**seconds between the digital output (yellow) and the start of the analog photodiode signal onsets for different triggering thresholds (blue = 180 and red = 10). The monitor was set to 100 brightness, 75 contrast, and ULMB mode was turned on.

CD and JM wrote the paper, designed and built the devices, ran tests and experiments, wrote the software, and designed the figures. CD drew the circuit diagrams in Figures 3, 4, and took photographs of the device for Figures 6–8. ST supported the project and helped proofread the paper.

### Conflict of Interest

The authors declare that the research was conducted in the absence of any commercial or financial relationships that could be construed as a potential conflict of interest. The handling editor declared a shared affiliation, though no other collaboration, with the authors at time of review.
